# Biographical Feature: In memory of Dennis Henry Bamford (1948–2025), who changed how we see viruses

**DOI:** 10.1128/jvi.00594-26

**Published:** 2026-06-03

**Authors:** Dave I. Stuart, Elizabeth E. Fry, Nicola G. A. Abrescia

**Affiliations:** 1 Division of Structural Biology, Centre for Human Genetics, Nuffield Department of Medicine, University of Oxford 6396, Oxford, United Kingdom; 2 Chinese Academy of Medical Sciences Oxford Institute, University of Oxford 6396, Oxford, United Kingdom; 3 Structure and Cell Biology of Viruses Laboratory, CIC bioGUNE, Basque Research and Technology Alliance (BRTA), Derio, Spain; 4 Ikerbasque, Basque Foundation for Science 197447, Bilbao, Spain; Indiana University Bloomington, Bloomington, Indiana, USA

## TEXT

Professor Dennis H. Bamford made groundbreaking contributions to molecular virology, revealing deep structural conservation across seemingly diverse groups of viruses. His work reshaped our understanding of virus evolution, transforming viral taxonomy and culminating in the recognition of the kingdom *Bamfordvirae*. A pioneer of integrative structural biology and scientific infrastructure, he combined insight, collaboration, and mentorship to influence and inspire generations of scientists. His legacy endures through his discoveries and the community he helped build.

## A NEW VISION OF THE VIRAL UNIVERSE

There are scientists who accumulate results and those who quietly change how a whole field thinks. Dennis Bamford did the latter, through careful work, insight, persistence, and humor. He dedicated his career to understanding the molecular basis of viruses, making seminal contributions to virus purification, crystallization, and, through longstanding collaborations, structural analysis using electron microscopy and X-ray diffraction. His work advanced not only methods and phage biology but also the fundamentals of how we understand the viral world.

**Fig 1:**
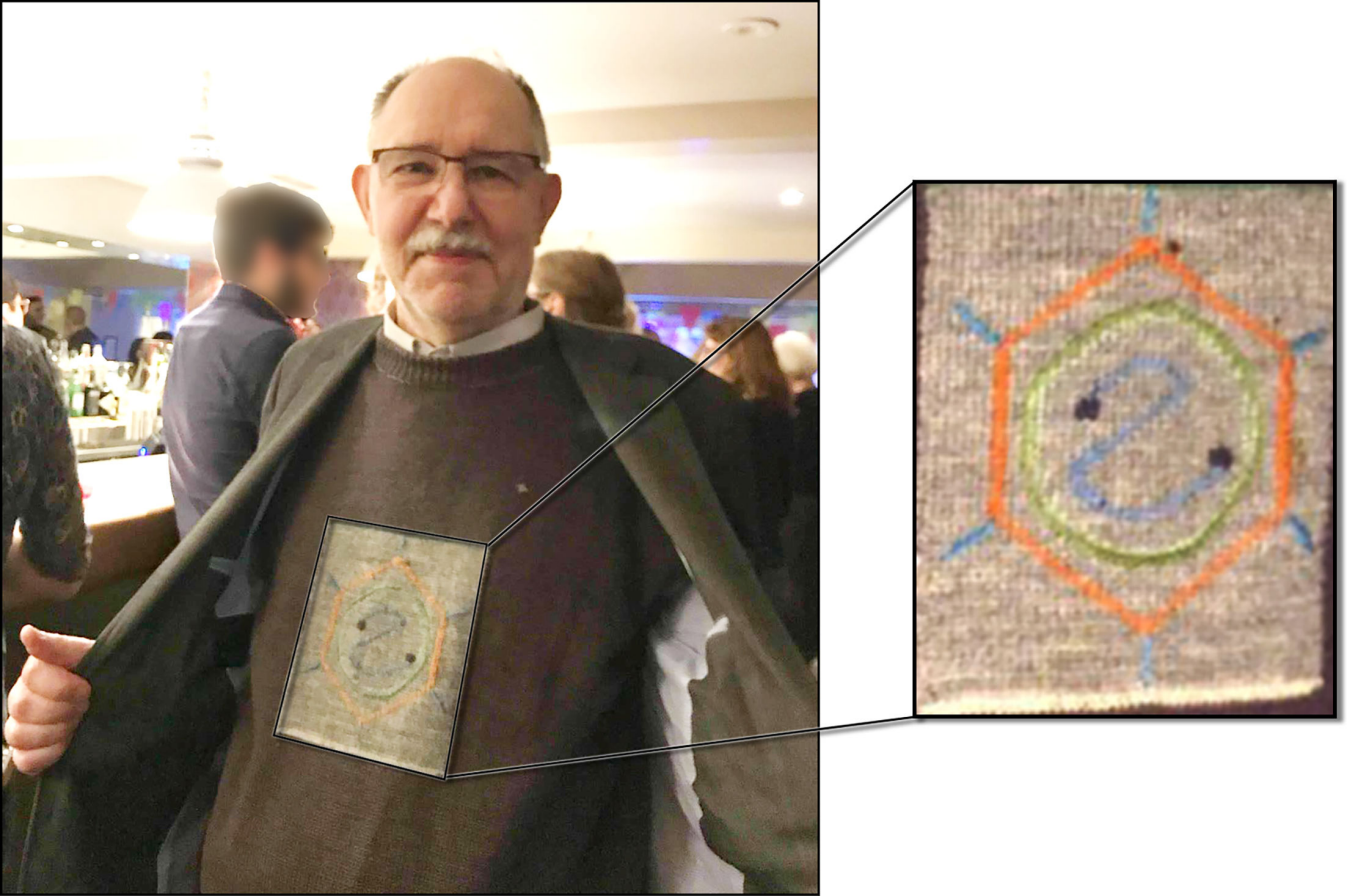
First low-resolution phi6 structure. Dennis’ most beloved jumper, featuring a schematic of phi6—he was always proud to wear it at meetings and conferences.

In a prelude to science, Dennis did military service in an artillery battery, joining the choir and cementing a lifelong friendship and a love of singing. His scientific career was spent almost entirely in Finland; however, a formative early experience was his time in New York with Leonard Mindich. There, work on the lipid-containing bacteriophage phi6 established a lasting interest in membrane-containing viruses and a long-standing collaboration. Phi6 remained central to his thinking and underpinned much later work (Fig. 1) (1–4).

One of Dennis’ key insights was that not only are the three-dimensional folds of viral proteins more conserved than their amino acid sequences but also that the 3D assembly puzzle of a virus capsid is a rare structure preserved over vast evolutionary timescales (5). The resulting constraints mean that core architectural features—what Dennis termed the virus “self”—remain recognizable long after sequence similarity is lost (6). This principle, demonstrated through studies of bacteriophage PRD1 and related viruses, provided a firm structural basis for understanding virus evolution at a much deeper level than had been possible using genome or amino acid sequences. The seminal high-resolution crystal structure of PRD1, the first virus with an internal lipid bilayer to be solved in atomic detail, established it as a model system for membrane-containing viruses and a platform for integrating biochemical, structural, and genetic approaches (7, 8).

Work at this highly productive time was supported by a Human Frontiers Science Programme grant (2001–2005), which brought together Roger Burnett, Stephen Fuller, and one of the authors (Dave Stuart). In the final report, Dennis wrote: “It is evident that the ideas that we have developed as a consequence of the HFSP fostered collaboration will have major consequences for virus taxonomy.”

The insight that structure could reveal otherwise inaccessible evolutionary relationships allowed Dennis to argue persistently that many virus families could be grouped into a small number of lineages based on conserved capsid-protein folds. He proposed that many viruses infecting bacteria, archaea, and eukaryotes shared common structural lineages—an idea seen initially as counterintuitive. Over time, accumulating structural evidence made the proposal increasingly compelling, and it is now reflected in the major viral lineages recognized today (9) and forms part of the modern framework for virus evolution and taxonomy (10, 11). Dennis’ contribution to this shift was ultimately recognized in the establishment of the viral kingdom *Bamfordvirae*. If we now view the virus world through a structural lens, it is in significant part because of this insight.

As noted above, Dennis’ contributions were rooted in collaboration. Across his career, he published nearly 400 peer-reviewed papers spanning virology, microbiology, and structural biology, many with international collaborators. He was deeply committed to supporting future generations, contributing extensively through book chapters and, as Editor-in-Chief of the fourth edition of the *Encyclopedia of Virology* (2021), shaping a comprehensive reference for the field (12). Even in that demanding setting, he retained his characteristic lightness: on at least one occasion, a particularly dry editorial meeting was rescued when Dennis broke into song.

## ARCHITECT OF INTEGRATIVE STRUCTURAL BIOLOGY

**Fig 2:**
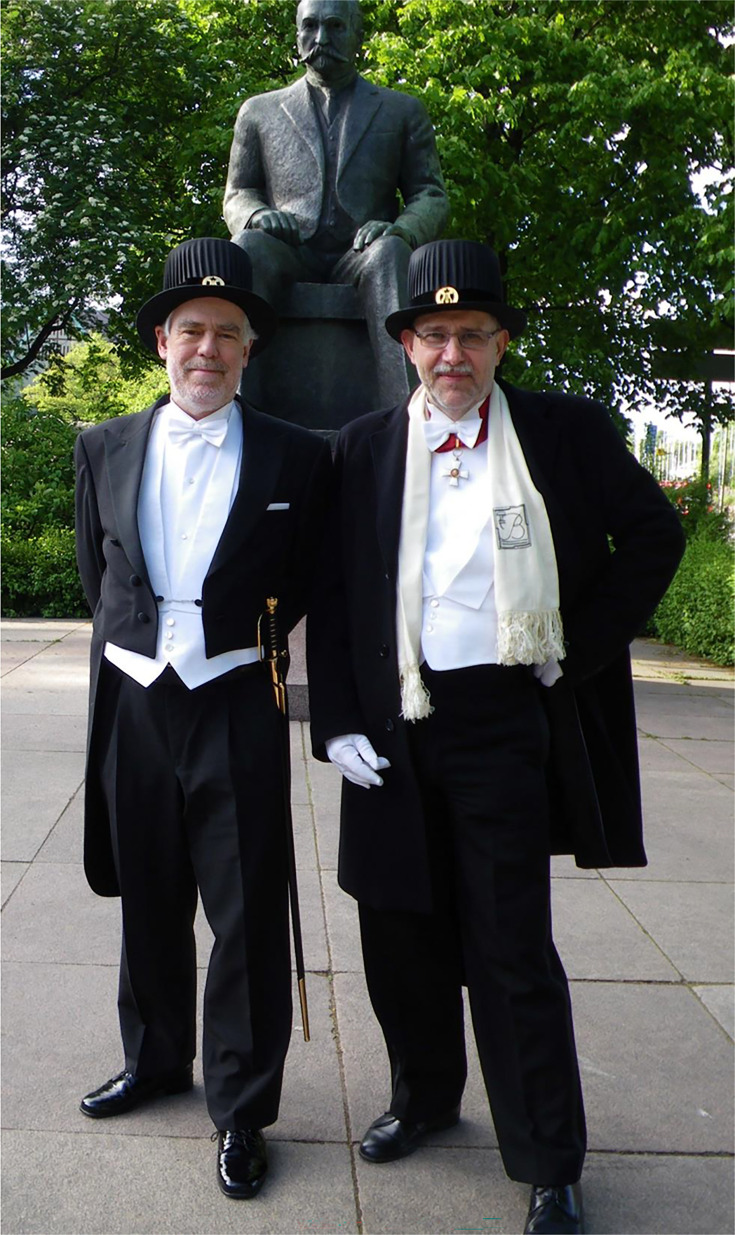
Sharing the vision of structural biology in Europe. Dennis and Dave Stuart (left) at the Honoris Causa conferment ceremony in Helsinki, wearing formal doctoral hats and holding doctoral swords (the man behind is Kyösti Kallio, the fourth President of the Republic of Finland).

Dennis’ broad vision led him to be an early advocate for interdisciplinary research, particularly integrating structural biology with virology to enable broader structural characterization of viruses. This led to a key role in developing Instruct-ERIC, the European infrastructure for structural biology (https://instruct-eric.org/). Working closely with colleagues including Dave Stuart, he helped shape Instruct as an open-access, collaborative resource (Fig. 2). He later served as Director of the Instruct Center for Virus Production in Helsinki, strengthening European capabilities in virus production and structural analysis. He was instrumental in bringing Finland into the consortium and strongly supported shared scientific infrastructure.

His contributions were widely recognized. He was twice appointed Academy Professor and led Center of Excellence programs funded by the Academy of Finland. In 2006, he was elected to the European Molecular Biology Organization, and in 2008, was appointed Commander of the Order of the Lion of Finland (although Dennis perhaps saw this as somewhat ironic).

## THE VALUE OF MENTORING

Dennis trained and influenced a generation of fine scientists who now lead independent research programs, including Martin Romantschuk, Mikko Frilander, Minna Poranen, Hanna Oksanen, Janne Ravantti, Erika Mancini, Mart Krupovic, Sarah Butcher, Juha Huiskonen, Eugene Makeyev, Nicola Abrescia, and Jonathan Grimes. Not only did his laboratory produce many independent scientists, but he was also instrumental in building community. Dennis brought the Phage/Virus Assembly (PVA) meeting to Europe for the first time in 2001 (https://pvaconference.wixsite.com/pva2025/about), establishing a tradition that continues to strengthen international links in virology.

**Fig 3:**
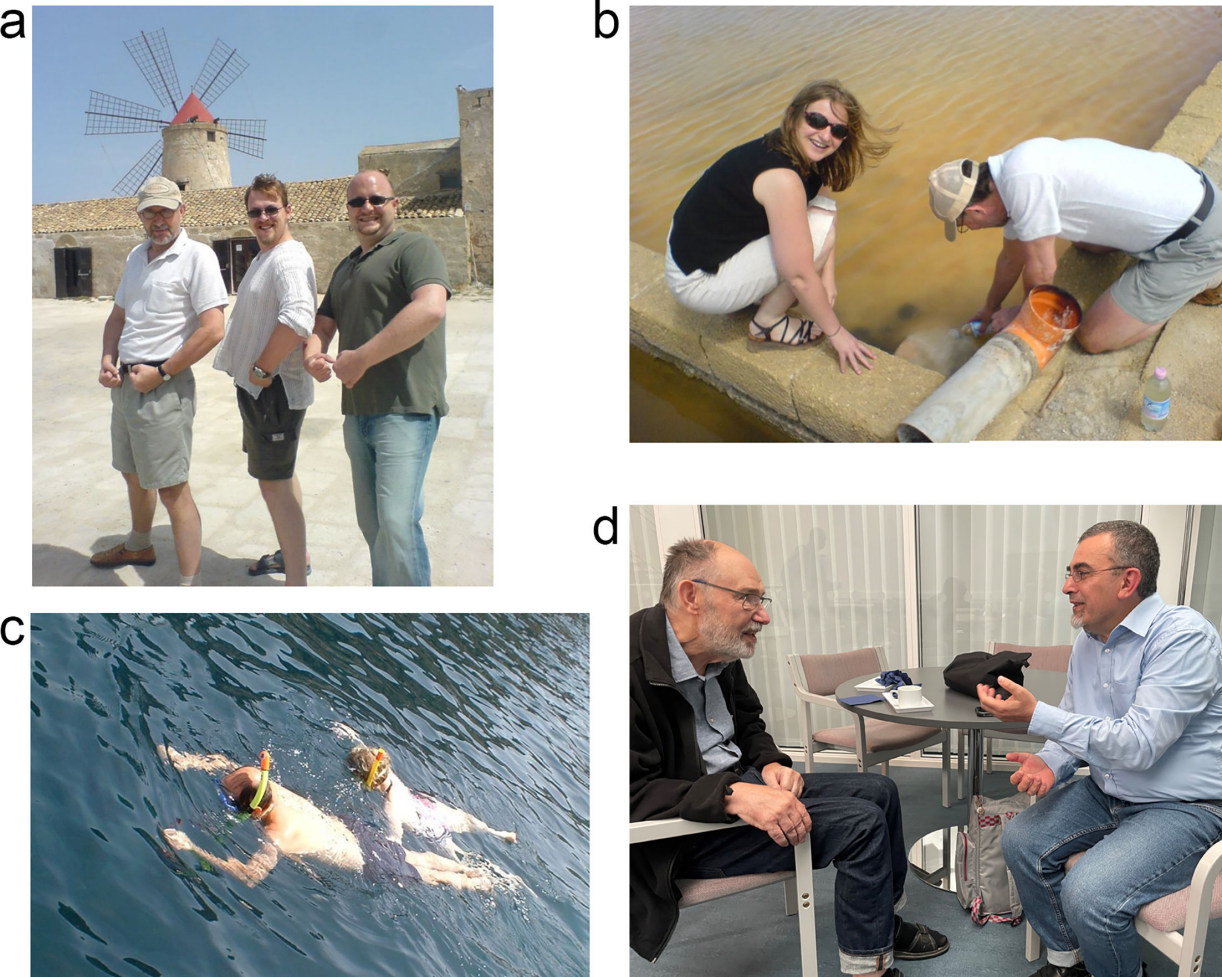
Exploring new ecological niches while mentoring in “different shapes and forms.” (**a**) Dennis with Tom Walter (center) and Christian Siebold (right) at the Trapani salt pans (Sicily). (**b**) Erika Mancini with Dennis, collecting a few centiliters of salt pan water. (**c**) Dennis with a team member, searching for phages and viruses in the blue fresh waters of San Vito Lo Capo (Sicily). (**d**) Mentoring never stops: Dennis asking questions after a seminar given by Nicola Abrescia at the University of Helsinki in August 2023 (pictured with Nicola Abrescia; photo by Juha Huiskonen).

Many of us remember his field expeditions—his “virus hunting” trips—across Europe. He led sampling campaigns from the salt pans of Sicily, including San Vito Lo Capo 2006 (Fig. 3a and b), to the coastal waters and salt works of Puglia in 2007, combining scientific curiosity with a genuine enjoyment of exploration, often at sea (Fig. 3c). These trips were not without moments of adventure—notably when, off Puglia, the boat’s steering wheel detached, leaving Dennis and crew drifting precariously. Fortunately, all ended well, and the expedition led to the isolation and later structural characterization of the salt-loving archaeal virus *Haloarcula hispanica* icosahedral virus 2 (13, 14). Such discoveries further extended the PRD1-related structural lineage and provided insights into viral assembly mechanisms.

Dennis’ mentoring style was light-touch but rigorous: he gave clear, direct feedback and encouraged independence. He remained engaged with former trainees long after they established their own groups, maintaining both scientific and warm personal connections (Fig. 3d). Connections stayed long after his retirement, in particular via Janne Ravantti, who gently liaised between Dennis and many of us out of Finland.

## CHARACTER AND LEGACY

Dennis was energetic, warm, engaging, generous, and always approachable, with a mischievous, irreverent sense of humor and a tendency to break into song. He showed absolute integrity, unwavering curiosity, and a deep commitment to collaboration and conversation, maintaining close international links, including longstanding connections to the United Kingdom. He remained an active scientist throughout his career, working at the bench as well as leading large initiatives. He had no airs or graces and expected to be respected for his science rather than his position and for his clarity of thought rather than past achievements. Dennis’ work changed how viruses are understood, showing that structure provides a unifying framework for the virosphere and that evolutionary relationships can be traced through architecture as well as sequence. His influence extends through both his discoveries and the many scientists he trained.

Professor Dennis Bamford will be deeply missed, but his impact will live on through the ideas he established and the community he helped build.
